# The Mediating Role of Oxidative Stress on the Association Between Oxidative Balance Score and Cancer-Related Cognitive Impairment in Lung Cancer Patients: A Cross-Sectional Study

**DOI:** 10.3390/nu16234090

**Published:** 2024-11-27

**Authors:** Xinxin Cheng, Lan Cheng, Jianyun He, Yuting Wang, Xiaoxia Lin, Shufang Xia

**Affiliations:** Wuxi School of Medicine, Jiangnan University, Wuxi 214122, China; 6222807009@stu.jiangnan.edu.cn (X.C.); chenglan@stu.jiangnan.edu.cn (L.C.); hejianyun@stu.jiangnan.edu.cn (J.H.); 6232807031@stu.jiangnan.edu.cn (Y.W.); 6232807016@stu.jiangnan.edu.cn (X.L.)

**Keywords:** cancer-related cognitive impairment, oxidative balance score, lung cancer, oxidative stress, diet, lifestyle

## Abstract

Objectives: To explore the association between the oxidative balance score (OBS) and cancer-related cognitive impairment (CRCI) in patients with lung cancer, as well as the oxidative stress biomarkers involved. Methods: In this cross-sectional study, 315 lung cancer patients were recruited, from whom 142 blood samples were collected to determine oxidative stress biomarkers. Dietary intake was assessed using 3-day, 24 h dietary recalls. The OBS was calculated by summing up pro- and antioxidant factors from a diet and lifestyles assessment. CRCI was evaluated using the Montreal Cognitive Assessment (MoCA) test. Results: A total of 103 patients (32.7%) developed CRCI, with significantly lower OBS and dietary OBS and lower superoxide dismutase (SOD) and glutathione peroxidase (GPx) activities than non-CRCI patients (*p* < 0.05). For every 1-point increase in OBS, the risk of CRCI was reduced by 10.6% (OR = 0.894; 95% CI 0.819, 0.977; *p* = 0.013). Both vitamin E (OR = 0.922; 95% CI 0.868, 0.980; *p* = 0.009) and dietary fiber (OR = 0.909; 95% CI 0.832, 0.992; *p* = 0.032) were significantly inversely related to CRCI. The association between the total OBS and CRCI was mediated by SOD (ACME = −0.0061; 95% CI −0.0170, −0.0004; *p* = 0.015) and GPx (ACME = −0.0069; 95% CI −0.0203, −0.0002; *p* = 0.032), respectively. Conclusions: Lung cancer patients with a greater balance of antioxidant to pro-oxidant diet, especially rich in dietary fiber and vitamin E, may decrease their CRCI in part by affecting SOD and GPx activities.

## 1. Introduction

Lung cancer is the most common type of cancer and the primary cause of cancer-related deaths worldwide [1]. During treatment, a wide range of symptoms relating to cancer, including chronic fatigue, dyspnea, psychological problems, and cognitive dysfunction, have been found to affect the health outcomes of lung cancer individuals [2]. Cancer-related cognitive impairment (CRCI), a common symptom in patients with lung cancer, had a prevalence ranging from 6% to 84.4% [3]. CRCI is conceptually defined as a condition of cognitive dysfunction experienced by cancer survivors, including problems with memory, attention, executive function, and information-processing speed. It can seriously affect daily functioning and quality of life [4]. Although CRCI is generally mild to moderate, it can persist for months or even years after treatment [5], leading to undesirable consequences such as depression and increased mortality [6], and is significantly associated with palliative treatment and unscheduled admission [7]. Nevertheless, there is limited evidence on modifiable CRCI risk factors in lung cancer patients.

A variety of studies have examined the various risk factors for CRCI, including aging, the disease itself and its treatment, socioeconomic factors, poor nutrition, smoking, and insufficient physical activity [4], of which lifestyle aspects are modifiable behavioral factors. As part of healthy lifestyles, a greater consumption of fruit and vegetable was positively associated with cognition among cancer patients [8], and increased monounsaturated fat and less saturated fat intake could be protective against CRCI during chemotherapy [9]. Conversely, unhealthy lifestyles (e.g., smoking, excessive alcohol consumption, physical inactivity, and high-fat and low-dietary-fiber diets) and being overweight were related with an elevated risk of cognitive decline [10,11]. Therefore, in order to provide targeted recommendations on lifestyles for the lung cancer patients to follow, it is necessary to confirm the association between lifestyle and CRCI.

Although the possible biological mechanisms linking lifestyle and CRCI are largely unknown, oxidative stress might be involved. Oxidative stress, a status of redox imbalance between pro-oxidants and antioxidants, can be caused by antineoplastic therapy and cancer itself [12], and leads to cognitive dysfunction [13]. Compared with other tissues, the brain is particularly vulnerable to oxidative stress and damage because of its high demand for oxygen, high levels of stored lipids, and relatively low concentrations of antioxidant enzymes [12]. Therefore, increasing the antioxidant capacity through healthy lifestyles, such as dietary antioxidants or physical activity, might alleviate oxidative stress from cancer and cancer treatment to delay or prevent cognitive decline. In a randomized controlled trial, an oral nutritional supplement containing antioxidant omega-3 polyunsaturated fatty acids (PUFA) has been reported to improve cognitive function in lung cancer patients [14]. A healthy dietary pattern, such as the Mediterranean diet with powerful antioxidant effects from high amounts of fruits, vegetables, and olive oil, can lead to better cognitive performance [15], while smoking, alcohol consumption, obesity, and dietary pro-oxidants contribute to systemic oxidative stress, resulting in an increased risk of cognitive decline [10,16]. Although studies on the role of diet on CRCI are limited, dietary nutrients (including folate, vitamin D, flavonoids, vitamin E, and certain lipids) or food groups (e.g., vegetables, sea food, and fruits) have been demonstrated to have antioxidant properties and have been associated with cognitive function in older adults [17], and therefore, these nutrients or food groups might influence the redox status and thus CRCI. Considering that the effect of a single lifestyle factor on CRCI is limited, a combined measure of various pro-oxidants and antioxidants may better reveal their relationship with CRCI. The oxidative balance score (OBS) represents the overall exposure to both pro-oxidants and antioxidants from an individual’s diet and lifestyle, with a higher OBS indicating higher antioxidant capacity [18]. The OBS was shown to be positively associated with cognitive performance in the elderly [19]. However, most research that explored the relationship between cognitive impairment and diet or lifestyles just focused on older community dwellers. To the best of our knowledge, there is no study that revealed an association between diet or lifestyles and CRCI in patients with lung cancer in the perspective of oxidative stress.

In fact, a variety of patients with lung cancer demonstrate an unhealthy dietary pattern, inadequate nutrient consumption, and tend to be sedentary due to the absence of professional guidance after disease diagnosis. Therefore, in order to provide more reliable dietary and lifestyle recommendations for lung cancer patients, this cross-sectional study was conducted to determine the relationship between the OBS and CRCI in patients with lung cancer and the roles of the oxidative stress biomarkers involved.

## 2. Materials and Methods

### 2.1. Participants and Study Design

This study (Chinese Clinical Trials Registry ChiCTR2300075273) was implemented at the Affiliated Hospital of Jiangnan University from September 2023 to May 2024. Three hundred and fifteen participants were selected based on these criteria: diagnosed with lung cancer without brain metastasis; aged 18 years or older; having a regular caregiver; voluntary participation. Patients were excluded based on the following criteria: diagnosis of other malignant tumors; prior physician-diagnosed mental illness or family history of mental illness; history of head trauma, cerebral hemorrhage, stroke, cerebral infarction; current use of psychiatric-related medications and dietary supplements; use of chemotherapy and radiotherapy within the last 21 days; incomplete clinical data or questionnaires; other reasons deemed unsuitable by the researchers. This research was approved by the Medical Ethics Committee of Jiangnan University (JNU20230601IRB09) and conducted in accordance with the Declaration of Helsinki. All participants provided their written informed consent before being enrolled.

### 2.2. Sample Size

Based on the formula $n=Z_{\alpha /2}^{2}\left(1-p\right)p/\delta^{2}$, in which α = 0.05, Z_α/2_ = 1.96, *p* was set at 0.26 based on the prevalence of CRCI among lung cancer patients [3], *δ* = 0.2, and *p* = 0.052. Consequently, the minimum sample size was estimated to be 274.

### 2.3. Assessment of Cognitive Function

The Montreal Cognitive Assessment (MoCA) test was used for assessment of cognitive function. The score of this tool is 0~30 with 7 dimensions: visual and executive ability, naming, attention, language, abstraction, memory and delayed recall, and orientation. A higher score indicates better cognitive performance. In the present study, CRCI was defined as a MoCA score of ≤13 (those with no formal education), ≤19 (those with 1~6 years of education), and ≤24 (those with 7 or more years of education) [20].

### 2.4. Physical Activity Assessment

In order to assess the intensity of physical activity, the International Physical Activity Questionnaire Short Form (IPAQ-SF) was used to calculate the metabolic equivalent (MET). Then, MET value × minutes of conducted physical activities during the day × days per week was calculated as a component for lifestyle OBS.

### 2.5. Dietary Intake Assessment

Dietary intake data were collected through 3-day, 24 h dietary recall interviews with the aid of food models and atlases. All patients and their caregivers were asked to recall in detail the types and amounts of foods the patients had consumed during the past 24 h prior to admission. Because most patients’ diets changed significantly during hospitalization, in order to collect the dietary intake data closer to patients’ daily lives, another two dietary recalls were collected on weekdays via WeChat after patients were discharged from the hospital. Considering the fact that most patients had a decreased appetite within a week after chemotherapy, we contacted patients and caregivers to confirm that the gastrointestinal symptoms had disappeared and then reminded them to record their diets. Patients or their caregivers weighed and photographed food, cooking oil, and spices using kitchen scales, and then sent the photos via WeChat to trained nutrition education research assistants, who in turn confirmed the patient’s food intake through video call. Nutrient intake was calculated using the Nutrition Calculator v2.8.1.8, and the average of the three dietary recalls data was used for statistical analysis.

### 2.6. OBS Calculation

Based on the association between oxidative stress and nutrients or lifestyle factors, the calculation of OBS was determined using 12 nutrients and 4 lifestyle factors, including 10 antioxidant and 6 pro-oxidant factors [18]. Data of lifestyle OBS components (smoking, alcohol use, obesity status, and physical activity) were collected. For smoking or alcohol use, never smokers or never drinkers received a score of 2; former smokers and former drinkers received a score of 1; and current smokers and current drinkers received a score of 0 [21]. For obesity status, the score for normal weight, overweight, and obese was 2, 1, and 0, respectively. Physical activity was assigned the scores 0–2 according to the tertile values corresponding to low (0), intermediate (1), and high (2). Dietary OBS components include dietary pro-oxidants, including saturated fatty acid (SFA), omega-6 fatty acids, and iron; and dietary antioxidants, including omega-3 fatty acids, monounsaturated fatty acid (MUFA), dietary fiber, vitamin C, vitamin E, vitamin A, β-carotene, selenium, and zinc. The dietary pro-oxidants were assigned scores of 0–2 according to the tertile values of each variable corresponding to low (2), intermediate (1), and high (0), whereas dietary antioxidants were assigned the scores 0–2 according to the tertile values of each variable corresponding to low (0), intermediate (1), and high (2). The detailed scoring criteria for each OBS component are shown in Table S1. Finally, the scores for each OBS component were summed to obtain a total OBS. A higher OBS indicates higher potential of antioxidant capacity.

### 2.7. Oxidative Stress Biomarkers Determination

Considering the effects of chemotherapy on individual’s redox status, we collected 142 fasting blood samples before chemotherapy after obtaining written informed consent from patients. The fresh blood was centrifuged at 3500 rpm for 10 min to separate plasma. Superoxide dismutase (SOD), GPx, glutathione (GSH), catalase (CAT), and malondialdehyde (MDA) were measured using the corresponding kits provided from Nanjing Jiancheng Bioengineering Institute (Nanjing, China).

### 2.8. Statistical Analyses

Statistical analyses were performed with SPSS 27.0 (IBM SPSS Inc., Chicago, IL, USA). Continuous variables are shown as mean ± standard deviation or median with 25th and 75th percentiles. Categorical variables are presented as number (*n*) and percentage (%). Differences between groups were analyzed using Mann–Whitney U test for non-normally distributed data indicated by the Kolmogorov–Smirnov test and categorical variables were analyzed using Chi-squared test or Fisher’s exact test. The association between OBS, dietary OBS, or oxidative stress biomarkers and CRCI was assessed using logistic regression analysis, while the association between OBS and oxidative stress biomarkers was analyzed using linear regression. Additionally, the potential mediating role of oxidative stress biomarkers on the association between OBS (exposure) and CRCI (outcome) was analyzed with R Mediation package 4.3.2. In this model, the average causal mediation effect (ACME, the indirect effect of OBS on CRCI through a biomarker of oxidative stress), average direct effect (ADE, the direct effect of OBS on CRCI excluding the oxidative stress biomarkers), and average total effect (ATE, the sum of ADE and ACME) were calculated. The 95% confidence interval (CI) was estimated using non-parametric bootstrapping with 5000 replications. All analyses were adjusted for age, sex, education level, residence, family monthly income, employment, pathology type, surgery, risk of malnutrition, and dietary energy intake. A value of *p* < 0.05 was considered statistically significant.

## 3. Results

### 3.1. Participants’ Characteristics

As shown in Table 1, among the 315 lung cancer patients, 103 (32.7%) patients were allocated to the CRCI group, while the remaining 212 (67.3%) patients were not suffering CRCI. Statistically significant differences were observed in age (*p* = 0.018), education level (*p* < 0.001), residence (*p* = 0.001), family monthly income (*p* < 0.001), surgery (*p* = 0.010), pathology subtype (*p* = 0.023), and risk of malnutrition (*p* = 0.001).

### 3.2. OBS Components

OBS components in the CRCI and non-CRCI groups are demonstrated in Table 2. Compared with the non-CRCI group, patients in the CRCI group had significantly decreased OBSs and dietary OBSs (both *p* < 0.001), and also had a remarkably lower intake of omega-6 fatty acids, omega-3 fatty acids, vitamin C, vitamin E, dietary fiber, β-carotene, iron, zinc, and selenium (*p* < 0.05).

### 3.3. Association Between OBS and CRCI

The correlations between the OBS, the dietary OBS, or its components and CRCI are illustrated in Table 3. After adjusting for age, sex, education level, residence, family monthly income, employment, pathology subtype, surgery, risk of malnutrition, and energy, when the OBS was set as a continuous variable, for every 1-point increase in the OBS, the risk of CRCI in lung cancer patients was reduced by 10.6% (OR = 0.894; 95% CI 0.819, 0.977; *p* = 0.013). When the OBS was set at tertiles, patients in the highest tertile of OBSs were 53.4% less likely to develop CRCI than patients in the lowest tertile of OBSs (OR = 0.466; 95% CI 0.221, 0.981; *p* for trend = 0.036). In addition, after adjusting for the same confounders described above, dietary OBS, vitamin E, and dietary fiber were all negatively associated with CRCI.

### 3.4. Plasma Oxidative Stress Biomarkers

**Figure 1:**
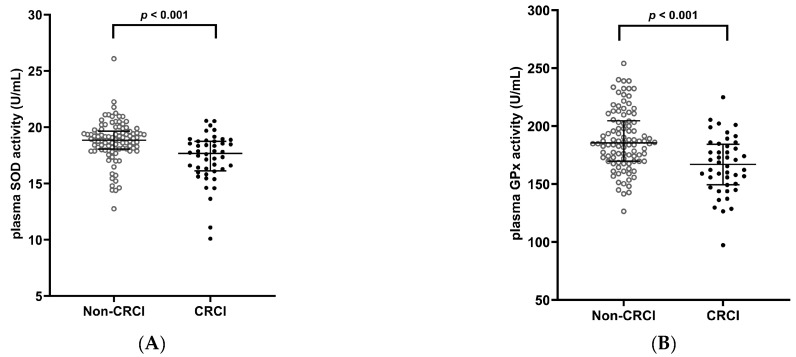

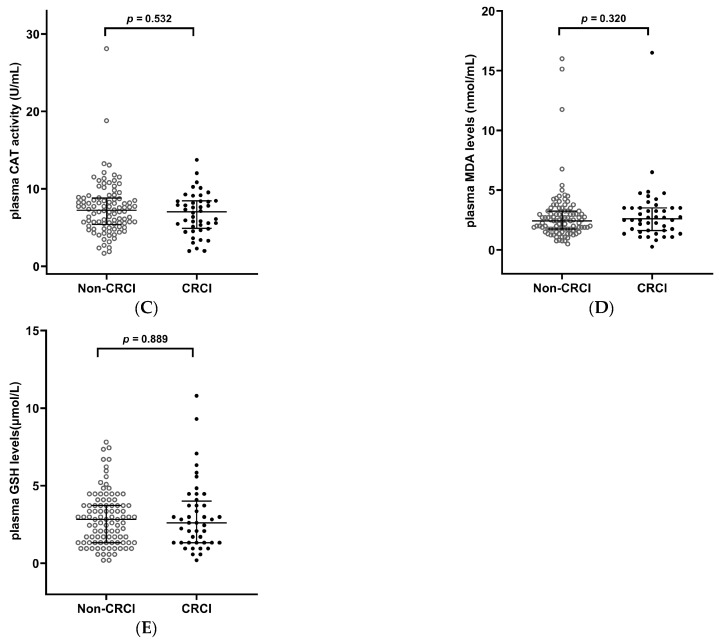
Oxidative stress biomarkers in CRCI and non-CRCI patients with lung cancer. (**A**) Plasma SOD activity; (**B**) Plasma GPx activity; (**C**) Plasma CAT activity, (**D**) Plasma MDA levels; (**E**) Plasma GSH levels. Data are shown as median (25th, 75th percentile). Mann–Whitney U test was used. CRCI, cancer-related cognitive impairment; SOD, superoxide dismutase; GPx, glutathione peroxidase; CAT, catalase; MDA, malondialdehyde; GSH, glutathione.

Plasma SOD and GPx activities were significantly lower in CRCI patients than in non-CRCI patients (*p* < 0.001, Figure 1A,B), but CAT, MDA, and GSH levels were not significantly different between them (*p* > 0.05, Figure 1C–E).

### 3.5. Associations Between Plasma Oxidative Stress Biomarkers and CRCI

As shown in Table S2, after adjusting for age, sex, education level, residence, family monthly income, employment, pathology subtype, surgery, risk of malnutrition, and energy, both SOD (OR = 0.632; 95% CI 0.499, 0.800; *p* < 0.001) and GPx (OR = 0.962; 95% CI 0.943, 0.982; *p* < 0.001) activities were negatively correlated with CRCI in lung cancer patients.

### 3.6. Association Between OBS and Plasma Oxidative Stress Biomarkers

As shown in Table S3, the OBS was positively related to SOD (β = 0.243; 95% CI 0.094, 0.323; *p* < 0.001) and GPx (β = 2.933; 95% CI 1.422, 4.444; *p* < 0.001).

### 3.7. Association Between OBS and CRCI with Oxidative Stress Biomarkers as Mediators

As shown in Table 4, results from the mediation analysis showed that the association between the OBS and CRCI was mediated by SOD (ACME = −0.0061; 95% CI −0.0170, −0.0004; *p* = 0.015) and GPx (ACME = −0.0069; 95% CI −0.0203, −0.0002; *p* = 0.032), respectively.

## 4. Discussion

This study demonstrated that both a lower OBS and a lower dietary OBS were correlated with CRCI in lung cancer patients, whereas both vitamin E and dietary fiber were negatively associated with CRCI. Furthermore, CRCI patients showed significantly lower SOD and GPx activities compared to non-CRCI patients. Both SOD and GPx activities played mediating roles in the association between the OBS and CRCI, suggesting that diet might affect CRCI through regulating the antioxidant capacity.

CRCI has become one of the most common symptoms among lung cancer patients. We found that CRCI was present in 32.7% of lung cancer patients, which was similar to the prevalence of 26.0–35.4% in previous studies [3,22]. A national cross-sectional study conducted in China reported that old age, rural residence, and fewer years of education were related with mild cognitive impairment [23]. In the present study, the average age of CRCI patients was 68.0 years, which was remarkably higher than the 66.0 years observed in non-CRCI patients, supporting the positive association of age with CRCI. Additionally, we also found that the CRCI patients were less educated, had a higher proportion living in rural areas and towns, and a lower monthly income. Commonly, patients who have less education and live in rural areas could not get a high income, better medical support, or healthy diet, which might decrease the brain’s cognition reserve [24] and contribute to the differences in cognitive function between CRCI and non-CRCI patients. Moreover, the different prevalence of CRCI in patients with SCLC and NSCLC might be related with the different cancer subtypes, as one study showed less structural damage to gray matter in patients with NSCLC than that in patients with SCLC [25]. It is noteworthy that surgery has been reported to lead to an increased risk of CRCI [4]. However, we found that the prevalence of CRCI was lower in lung cancer patients who underwent surgery than in those who did not. This difference might be attributed to the progression of the disease, as patients with early-stage lung cancer are mainly treated by surgery, whereas patients with advanced stage are inoperable and they have to tolerate the neurotoxic effects of systemic chemotherapy. Therefore, healthcare professionals should prioritize lung cancer patients who are old, less educated, have lower incomes, live in rural areas, and are diagnosed with advanced-stage lung cancer to prevent or treat CRCI.

Although the food frequency questionnaire (FFQ) is commonly used to assess the long-term diet of an individual, we used the 3-day, 24 h dietary recall method instead. The reason for this change was that we found that most of the lung cancer patients’ dietary habits had changed a lot after the diagnosis of the disease, and the diet information collected using the FFQ included both dietary intake before and after the diagnosis, so it was not possible to reflect the diets of the patients in the disease stage only. A variety of dietary elements and lifestyles have been reported to have an impact on cognitive performance in non-cancer populations [26]. Based on the NHANES 2011–2014, a number of publications have reported the associations between different dietary redox status indexes or physical activity and the risk of cognitive impairment in older adults. For example, lower scores on the Composite Dietary Antioxidant Index based on dietary vitamin A, C, E, zinc, selenium, and carotenoids, as well as a lack of physical activity, have been shown to be associated with cognitive impairment in the elderly [27]. The total antioxidant capacity (TAC), calculated from eight dietary antioxidant vitamins, was also negatively correlated with cognitive impairment in the elderly, especially diabetic patients at risk for oxidative damage [28]. When the TAC was calculated based only on dietary intakes of carotenoids, flavonoids, vitamin C, and vitamin E, it was correlated with a reduced odds of cognitive dysfunction in later life [29]. Similarly, the OBS has been found to have a positive correlation with cognitive performance in older adults [19]. However, there are fewer studies addressing individual antioxidant capacity and CRCI. The reality is that cancer survivors often have a desire to obtain professional dietary recommendations to improve their health and alleviate the ongoing effects of the disease and treatment [30]. Survivors have been reported to believe that diet affects their thinking ability, and some survivors change their diet to improve cognitive performance [31]. Here, we observed that the dietary OBS was inversely correlated with CRCI in lung cancer patients. Among dietary OBS components, only vitamin E and dietary fiber were significantly different between CRCI and non-CRCI patients, and both were negatively associated with CRCI. Vitamin E, a powerful antioxidant, is useful in improving cognition. Both the Shanghai Aging cohort study and the Singapore Chinese Health Study demonstrated the negative associations between vitamin E intake and the risk of dementia [32] or cognitive impairment in older adults [29]. Similarly, among 14,968 U.S. women in the prospective cohort of the Nurses’ Health Study, women who took a combination of vitamin E and C supplements over a long period of time had a slower cognitive decline, which was strongest among participants with a lowest dietary vitamin E intake at baseline [33]. The positive role of dietary fiber in cognitive function in older adults has also been reported. A 13-year cohort of older women demonstrated that participants with a low dietary fiber intake had a more significant cognitive decline [34]. In addition, a cohort study showed significant improvements in cognitive function over a 10-year period in participants aged 50 and older with high dietary fiber intake [35]. Data from NHANES cohort also showed a strong positive correlation between dietary fiber and cognitive function in the elderly [36]. However, the dietary fiber intake of Chinese residents has continued to decline since 1982. By 2015, the per capita daily intake of dietary fiber among Chinese adults had dropped to 9.7 g [37]. Here, the dietary fiber intake of CRCI and non-CRCI patients was only 5.6 and 7.6 g/per capita/d, respectively, both of which were lower than the dietary fiber intake of the general population. Therefore, health interventions aimed at increasing dietary vitamin E and fiber intake (e.g., through increased intake of coarse grains, vegetables, and fruits) may combat CRCI in lung cancer patients by increasing the dietary OBS.

In this study, no significant correlation was found between the lifestyle OBS and CRCI. The four lifestyle OBS components, including smoking status, alcohol use, obesity status, and physical activity, did not differ significantly between CRCI and non-CRCI patients. A previous study also failed to reveal remarkable association between smoking status and cognitive performance in lung cancer participants [38]. We speculated that the reason might be that most lung cancer patients would actively adjust their unhealthy lifestyles, such as quitting smoking and alcohol after the diagnosis of the disease. A clinical trial suggested that physical activity had an ameliorating effect on brain structure changes in lung cancer patients due to systemic chemotherapy and brain radiation [25]. Although a variety of studies revealed that physical activity has associations with cognitive function, in which physical activity was determined using direct observation or objective assessment and indirect or subjective assessment, we failed to observe the significant association between them, possibly because the IPAQ-SF we used was relatively simple and did not investigate the intensity and type of the physical activity in detail. Given the complex nature of physical activity, none of the currently available methods could assess all physical activity dimensions, including the duration, frequency, intensity, and type. The reality is that most lung cancer survivors do not meet physical activity guidelines due to the effects of surgery and chemotherapy, possibly leading to a reduced sensitivity of the IPAQ-SF for the assessment of physical activity. In the future, we can use both subjective physical activity questionnaires and objective assessments using accelerometers to monitor different dimensions of physical activity, which might help us to reveal the real association between physical activity and CRCI.

Growing evidence suggests that oxidative stress is a major contributor to CRCI [39]. It is well-known that the brain consumes about 20% of the body’s total oxygen supply and is particularly susceptible to oxidative stress. Oxidative stress biomarkers include oxidants (e.g., reactive oxygen species), oxidative stress metabolites (e.g., MDA), antioxidant enzymes (e.g., SOD, CAT, GPx), and non-enzymatic antioxidants (e.g., GSH) [40], among which antioxidant enzymes are the first line of defense in the antioxidant defense system and are represented by SOD, CAT, and GPx, which scavenge hydrogen peroxide and lipid hydroperoxides. Individuals with mild cognitive impairment had decreased levels of GSH and GPx, and increased lipid peroxidation compared with controls [41]. A healthy diet is a very important source of antioxidants, and the consumption of plant-derived foods, which contain dietary fiber, vitamins, and phytochemicals, may increase the activity of SOD and GPx, which inhibit oxidative stress and thereby maintain normal cognitive function [42]. Animal studies have revealed that the number of hippocampal neurons degenerating after the administration of anti-cancer agents was associated with diminished activity of antioxidant enzymes and increased lipid peroxidation levels [43]. Notably, older adults who were less physically active may reduce the risk of cognitive impairment by increasing their intake of antioxidant-rich foods [27]. Previous observational and experimental studies have demonstrated that certain healthy dietary and exercise regimens can mitigate oxidative stress by regulating antioxidant enzymes (e.g., CAT, SOD, GPx) to decrease MDA levels, thereby enhancing cognitive function [44,45]. Because of the instability of reactive oxygen species, oxidants determination is commonly tested in animal experiments. Considering that the plasma volume after centrifugation was limited, we chose SOD, GPx, GSH, CAT, and MDA as the oxidative stress biomarkers based on the above literature. In this research, CRCI patients had lower plasma SOD and GPx activities than non-CRCI patients, and both of these two enzymes were mediators of the association between OBS and CRCI. Future research can further determine other oxidative stress biomarkers, such as glutathione S-transferase, sulfhydryl oxidase, and peroxiredoxin, to reveal more mediators in the association and also perform chain mediation analysis to explore the association between different oxidative stress biomarkers. Therefore, a healthy diet rich in antioxidants, especially vitamin E and dietary fiber, may contribute to cognitive improvement in lung cancer patients through increasing antioxidant capacity.

Despite the strengths of this study, there are some limitations. First, this was a cross-sectional study and it was not possible to determine the temporal order and causality between OBS, oxidative stress biomarkers, and CRCI. Second, patients in the dietary survey were prone to recall bias. Third, as a single-center study, the enrolled patients might not be fully representative of the patient population, the generalizability of the findings to other populations might be limited. Finally, it is very common for lung cancer patients to consume inadequate food, excluding the possibility of excessive pro-oxidants and antioxidants intake. Therefore, threshold effects of antioxidants were not taken into account in the calculation of the OBS. Furthermore, all OBS components were weighted equally in the overall score, which might obscure the individual contributions of different dietary and lifestyle factors. Therefore, it is necessary to design prospective studies with follow-up to further reveal the association between diet, lifestyle, and CRCI.

## 5. Conclusions

Our findings illustrated that the OBS was inversely related to CRCI in lung cancer patients, possibly in part by increasing SOD and GPx activities. The dietary OBS and its components, dietary fiber and vitamin E, were all significantly associated with CRCI. Therefore, a diet rich in antioxidants could be a promising way to reduce systemic oxidative stress and prevent or mitigate CRCI. Lung cancer patients should be encouraged to increase the intake of antioxidant foods such as vegetables, fruits, whole grains, MUFA-rich olive oil, and PUFA-rich sea food, as well as reduce the intake of pro-oxidant foods, such as red meat and processed meats rich in saturated fats, to increase the antioxidant capacity and thereby improve the quality of life of lung cancer patients through dietary modifications to improve their cognitive function. We should also conduct further prospective studies to clarify the causal association between OBS and CRCI to compensate for the shortcomings of the present cross-sectional study.

## Figures and Tables

**Table 1:** Characteristics of the CRCI and Non-CRCI patients with lung cancer.

	CRCI (*n* = 103)	Non-CRCI (*n* = 212)	*p*-Value
Age (years) ^1^	68.0 (63.0, 72.0)	66.0 (60.0, 71.0)	0.018
Sex, *n* (%) ^2^			
Male	79 (76.7)	154 (72.6)	0.441
Female	24 (23.3)	58 (27.4)	
Marital status, *n* (%) ^2^			
Widowed/divorced/single	7 (6.8)	13 (6.1)	0.821
Married	96 (93.2)	199 (93.9)	
Education level, *n* (%) ^2^			
Primary school or lower	39 (37.9)	57 (26.9)	<0.001
Middle school	56 (54.3)	90 (42.4)	
High school and above	8 (7.8)	65 (30.7)	
Employment, *n* (%) ^2^			
Employed	3 (2.9)	16 (7.5)	0.058
Unemployed	14 (13.6)	15 (7.1)	
Retired	86 (83.5)	181 (85.4)	
Residence, *n* (%) ^2^			
Rural areas	40 (38.8)	43 (20.3)	0.001
Towns	13 (12.6)	22 (10.4)	
Urban areas	50 (48.6)	147 (69.3)	
Family monthly income (RMB), *n* (%) ^2^			
<3000	31 (30.1)	26 (12.3)	<0.001
3000–5000	51 (49.5)	76 (35.8)	
>5000	21 (20.4)	110 (51.9)	
Presence of comorbidities, *n* (%) ^2^			
No	49 (47.6)	113 (53.3)	0.340
Yes	54 (52.4)	99 (46.7)	
Surgery, *n* (%) ^2^			
No	65 (63.1)	101 (47.6)	0.010
Yes	38 (36.9)	111 (52.4)	
Pathology subtype, *n* (%) ^2^			
Non-small cell lung cancer	86 (83.5)	195 (92.0)	0.023
Small cell lung cancer	17 (16.5)	17 (8.0)	
Cancer stage, *n* (%) ^2^			
I	12 (11.7)	33 (15.6)	0.670
II	8 (7.8)	19 (9.0)	
III	22 (21.3)	49 (23.0)	
IV	61 (59.2)	111 (52.4)	
Risk of malnutrition, *n* (%) ^2^			
No	78 (75.7)	190 (89.6)	0.001
Yes	25 (24.3)	22 (10.4)	
Chemotherapy cycle, *n* (%) ^2^			
T0~T1	53 (51.5)	100 (47.2)	0.751
T2~T6	32 (31.1)	74 (34.9)	
≥T7	18 (17.4)	38 (17.9)	
Energy intake (kcal/d) ^1^	1006.0 (828.0, 1258.0)	1134.0 (962.3, 1383.0)	0.002

Data are shown as *n* (%) or median (25th, 75th percentile). ^1^ Mann–Whitney U test; ^2^ Chi-squared test. CRCI, cancer-related cognitive impairment.

**Table 2:** OBS and its components in CRCI and Non-CRCI patients with lung cancer.

	CRCI (*n* = 103)	Non-CRCI (*n* = 212)	*p*-Value
OBS ^1^	16.0 (14.0, 19.0)	18.0 (16.0, 21.0)	<0.001
Lifestyle OBS ^1^	5.0 (4.0, 6.0)	5.5 (5.0, 6.0)	0.411
Dietary OBS ^1^	11.0 (8.0, 13.0)	13.0 (10.0, 15.0)	<0.001
Lifestyle OBS components			
Smoking status, *n* (%) ^2^			
Never	29 (28.1)	80 (37.7)	0.239
Former	69 (67.0)	122 (57.6)	
Current	5 (4.9)	10 (4.7)	
Alcohol use, *n* (%) ^3^			
Never	47 (45.6)	116 (54.7)	0.157
Former	55 (53.4)	90 (42.5)	
Current	1 (1.0)	6 (2.8)	
Obesity status, *n* (%) ^2^			
Obese	6 (5.8)	19 (9.0)	0.274
Overweight	31 (30.1)	48 (22.6)	
Normal weight	66 (64.1)	145 (68.4)	
Physical activity ^1^	696.0 (198.0, 1396.5)	696.0 (342.0, 1393.5)	0.689
Dietary OBS components			
SFA (g/d) ^1^	11.2 (8.3, 14.8)	12.4 (8.7, 17.9)	0.110
Omega-6 fatty acids (mg/d) ^1^	549.4 (391.7, 730.1)	631.6 (479.6, 888.7)	0.003
Iron (mg/d) ^1^	12.5 (9.5, 15.7)	14.2 (10.8, 17.5)	0.011
Omega-3 fatty acids (mg/d) ^1^	87.5 (60.4, 110.7)	99.3 (70.6, 132.0)	0.021
MUFA (g/d) ^1^	12.7 (8.6, 18.5)	13.7 (9.3, 19.6)	0.238
Dietary fiber (g/d) ^1^	5.6 (3.6, 7.4)	7.6 (5.7, 10.7)	<0.001
Vitamin C (mg/d) ^1^	64.0 (35.6, 104.3)	87.5 (48.1, 134.6)	0.003
Vitamin E (mg/d) ^1^	9.1 (6.6, 11.7)	11.7 (8.2, 15.2)	<0.001
Vitamin A (µgRAE/d) ^1^	355.0 (213.0, 508.0)	389.5 (280.3, 526.3)	0.062
β-Carotene (µg/d) ^1^	928.5 (462.6, 1782.0)	1393.9 (887.8, 2317.2)	0.001
Zinc (mg/d) ^1^	7.4 (5.9, 9.3)	8.4 (6.3, 10.6)	0.005
Selenium (µg/d) ^1^	32.6 (22.5, 48.9)	40.8 (26.5, 59.5)	0.008

Data are shown as *n* (%) or median (25th, 75th percentile). ^1^ Mann–Whitney U test; ^2^ Chi-squared test; ^3^ Fisher’s exact test. CRCI, cancer-related cognitive impairment; OBS, oxidative balance score; SFA, saturated fatty acid; MUFA, monounsaturated fatty acid.

**Table 3:** Association between OBS and CRCI in patients with lung cancer (*n* = 315).

	OR	95% CI	*p*-Value
OBS (continuous)	0.894	0.819, 0.977	0.013
Dietary OBS	0.882	0.801, 0.971	0.011
OBS (categorical)			
Lowest tertile (8 to 16)	1.000	Reference	0.036 *
Middle tertile (17 to19)	0.635	0.331, 1.217	
Highest tertile (20 to 27)	0.466	0.221, 0.981	
OBS components			
Omega-6 fatty acids	0.999	0.998, 1.000	0.141
Iron	0.989	0.942, 1.037	0.640
Omega-3 fatty acids	0.999	0.994, 1.003	0.564
Dietary fiber	0.909	0.832, 0.992	0.032
Vitamin C	0.999	0.994, 1.004	0.588
Vitamin E	0.922	0.868, 0.980	0.009
β-Carotene	1.000	1.000, 1.000	0.224
Zinc	0.943	0.847, 1.049	0.277
Selenium	0.996	0.987, 1.005	0.345

Multivariate logistic regression was used after adjusting for age, sex, education level, residence, family monthly income, employment, pathology subtype, surgery, risk of malnutrition, and energy. The association between dietary OBS and CRCI was additionally adjusted for lifestyle OBS. * *p*-value for trend derived using the median approach. OBS, oxidative balance score; CRCI, cancer-related cognitive impairment.

**Table 4:** Mediating effects of oxidative stress biomarkers on the association between OBS and CRCI in patients with lung cancer (*n* = 142).

Mediators	ATE			ADE			ACME		
	Estimate	95% CI	*p*-Value	Estimate	95% CI	*p*-Value	Estimate	95% CI	*p*-Value
SOD	−0.0155	−0.0241, −0.0028	0.010	−0.0094	−0.0190, 0.0022	0.072	−0.0061	−0.0170, −0.0004	0.015
GPx	−0.0150	−0.0243, −0.0023	0.008	−0.0081	−0.0179, 0.0040	0.096	−0.0069	−0.0203, −0.0002	0.032

Mediation analysis was used after adjusting for age, sex, education level, residence, family monthly income, employment, pathology subtype, surgery, risk of malnutrition, and energy. The 95% CI was computed from 5000 non-parametric bootstrap replicates. ATE, average total effect; ADE, average direct effect; ACME, average causal mediation effect; 95% CI, 95% confidence interval; OBS, oxidative balance score; SOD, superoxide dismutase; GPx, glutathione peroxidase; CRCI, cancer-related cognitive impairment.

## Data Availability

The data presented in this study are available from the corresponding author upon request due to privacy.

## Supplementary Materials

The following supporting information can be downloaded at: https://www.mdpi.com/article/10.3390/nu16234090/s1, Table S1: Oxidative balance score (OBS) assignment scheme; Table S2: Association between oxidative stress biomarkers and CRCI in lung cancer patients (*n* = 142); Table S3: Association between OBS and oxidative stress biomarkers in lung cancer patients (*n* = 142).
