# Supplementary material for: The Mediating Role of Oxidative Stress on the Association Between Oxidative Balance Score and Cancer-Related Cognitive Impairment in Lung Cancer Patients: A Cross-Sectional Study
Source: Nutrients. 2024 Nov 27;16(23):4090. doi: 10.3390/nu16234090 (PMC11643973; doi:10.3390/nu16234090)
Supplement: Supplementary file 1 [file nutrients-16-04090-s001.zip › Supplementary table.pdf]

Table S1. Oxidative balance score (OBS) assignment scheme.

OBS Components	Assignment Scheme
Lifestyle OBS components	
Smoking (P)	0 = current smoker, 1 = former smoker, 2 = never smoker
Alcohol use (P)	0 = current drinker, 1 = former drinker, 2 = never drinker
Obesity status (P)	0 = obese, 1 = overweight, 2 = normal
Physical activity (A)	0 = low (1st tertile), 1 = intermediate (2nd tertile), 2 = high (3rd tertile)
Dietary OBS components	
SFA (P)	0 = high (3rd tertile), 1 = intermediate (2nd tertile), 2 = low (1st tertile)
Omega-6 fatty acids (P)	0 = high (3rd tertile), 1 = intermediate (2nd tertile), 2 = low (1st tertile)
Iron (P)	0 = high (3rd tertile), 1 = intermediate (2nd tertile), 2 = low (1st tertile)
Omega-3 fatty acids (A)	0 = low (1st tertile), 1 = intermediate (2nd tertile), 2 = high (3rd tertile)
MUFA (A)	0 = low (1st tertile), 1 = intermediate (2nd tertile), 2 = high (3rd tertile)
Dietary fiber (A)	0 = low (1st tertile), 1 = intermediate (2nd tertile), 2 = high (3rd tertile)
Vitamin C (A)	0 = low (1st tertile), 1 = intermediate (2nd tertile), 2 = high (3rd tertile)
Vitamin E (A)	0 = low (1st tertile), 1 = intermediate (2nd tertile), 2 = high (3rd tertile)
Vitamin A (A)	0 = low (1st tertile), 1 = intermediate (2nd tertile), 2 = high (3rd tertile)
β-carotene (A)	0 = low (1st tertile), 1 = intermediate (2nd tertile), 2 = high (3rd tertile)
Selenium (A)	0 = low (1st tertile), 1 = intermediate (2nd tertile), 2 = high (3rd tertile)
Zinc (A)	0 = low (1st tertile), 1 = intermediate (2nd tertile), 2 = high (3rd tertile)

P, pro-oxidant; A, antioxidant; SFA, saturated fatty acid; MUFA, monounsaturated fatty acid.

Table S2. Association between oxidative stress biomarkers and CRCI in lung cancer patients (*n* =142).

	**OR**	**95% CI**	***p*-Value**
SOD	0.632	0.499, 0.800	< 0.001
GPx	0.962	0.943, 0.982	< 0.001

Multivariate logistic regression was used after adjusting for age, sex, education level, residence, family monthly income, employment, pathology subtype, surgery, risk of malnutrition, and energy. 95% CI, 95% confidence interval; OR, odds ratio; SOD, superoxide dismutase; GPx, glutathione peroxidase.

Table S3. Association between OBS and oxidative stress biomarkers in lung cancer patients (n = 142).

	**SOD**			**GPx**		
	**β**	**95% CI**	***p*-Value**	**β**	**95% CI**	***p*-Value**
OBS	0.243	0.094, 0.323	< 0.001	2.933	1.422, 4.444	< 0.001

Data were shown as beta coefficient and 95% confidence interval. Multiple linear regression was used after adjusting for age, sex, education level, residence, family monthly income, employment, pathology subtype, surgery, risk of malnutrition and energy. 95% CI, 95% confidence interval; OBS, oxidative balance score; SOD, superoxide dismutase; GPx, glutathione peroxidase.
